# Clinical symptoms and prognosis of patients with pulmonary nodules: a real-world cohort study

**DOI:** 10.3389/fmed.2026.1932201

**Published:** 2026-09-09

**Authors:** Shiting Zhou, Ling Song, Wei Zhang, Chunxia Yang, Bei Zhao, Jun Chen

**Affiliations:** Department of Respiratory and Critical Care Medicine, Zhejiang Provincial People's Hospital Bijie Hospital, Bijie, Guizhou, China

**Keywords:** CIS fatigue, clinical symptoms, post-COVID-19, prognosis, pulmonary nodules

## Abstract

**Objective:**

To identify risk factors for pulmonary nodule progression based on a prospective cohort of 392 patients.

**Methods:**

Baseline clinical, imaging and quantitative indicators including volume doubling time (VDT) and fatigue scores were compared between patients with stable and progressive nodules. Univariate and multivariate Cox regression models were used to analyze prognostic factors.

**Results:**

Among all subjects, 61.0% had COVID-19 infection history, and 33.7% of nodules presented lobulation and spiculation. During follow-up, 25.2% of nodules progressed, and the progression group had shorter VDT and higher fatigue scores (*P* < 0.05). Nevertheless, CIS-Fatigue could not predict the progression of pulmonary nodules. Multivariate Cox regression demonstrated that age, smoking history and irregular nodule margins were independent risk factors. Each 5-year age increase and smoking history raised progression risk by 1.2-fold. Nodules with spiculated and lobulated margins had a 1.5-fold higher growth risk, while COVID-19 infection was not an independent predictor.

**Conclusion:**

Age, smoking status and high-risk imaging features predict nodule progression. Risk-stratified follow-up should be performed for high-risk populations.

## 1 Introduction

As the main cause of cancer death in the world, lung cancer is still a significant public health burden worldwide (1–3). In recent years, with the widespread application of low-dose computed tomography (LDCT) in early lung cancer screening and physical examinations, the detection rate of pulmonary nodules has increased significantly year by year, which has become one of the most common thoracic lesions in clinical practice of respiratory medicine, thoracic surgery and imaging department (4–6). Pulmonary nodules are round or irregular pulmonary lesions with a diameter of no more than 3 cm on imaging manifestations, which can be divided into solid, part-solid and ground-glass nodules. Their pathological types include benign lesions such as inflammatory lesions, pulmonary fibrosis and tuberculoma, as well as malignant lesions including adenocarcinoma *in situ*, minimally invasive adenocarcinoma and invasive lung cancer (7–11). Due to complex etiology, highly heterogeneous imaging manifestations and lack of specific clinical manifestations in early malignant nodules, clinical practice is faced with excessive examination and intervention for low-risk benign nodules, as well as insufficient identification and delayed diagnosis and treatment of high-risk malignant nodules, bringing great challenges to clinical stratified management and individualized diagnosis and treatment.

Clinical symptoms serve as important bases for disease screening, condition evaluation and prognosis judgment. Previous studies have confirmed that most patients with early pulmonary nodules have no typical symptoms and are mostly detected incidentally during physical examinations or examinations for other diseases. Only patients with large nodules, special lesion locations or malignant tendency may present respiratory symptoms such as cough, expectoration, chest distress, chest pain and hemoptysis (10, 12). Nevertheless, large-sample and long-term follow-up real-world data focusing on the distribution characteristics of clinical symptoms of pulmonary nodules and their correlation with imaging features and malignant risk are still insufficient. Meanwhile, the long-term prognosis of patients with pulmonary nodules is affected by multiple factors including age, nodule size, density, morphology, clinical symptoms and pathological nature. Most existing studies focus on lung cancer high-risk population screening or differential diagnosis of benign and malignant nodules, while real-world cohort studies exploring the correlation between clinical symptoms and long-term prognosis in the general population of pulmonary nodules are relatively scarce, which cannot fully guide clinical individualized risk assessment and whole-process management strategy formulation.

Real-world research is conducted based on actual clinical scenarios. Its data are more consistent with clinical practice, which can objectively reflect the real clinical characteristics and prognostic outcomes of diseases, and provide more practical evidence-based references for clinical decision-making (13, 14). Therefore, this study enrolled clinically diagnosed patients with pulmonary nodules to establish a real-world cohort, systematically analyzed the distribution characteristics of their clinical symptoms, explored the correlation between symptomatic characteristics, imaging indicators and malignant risk of pulmonary nodules, and further followed up to evaluate long-term prognosis and influencing factors. This study aims to provide evidence for early identification of high-risk groups, clinical stratified management and optimization of individualized diagnosis and treatment strategies for pulmonary nodules.

## 2 Methods

### 2.1 Study design

This was a single-center, prospective, observational cohort study designed to investigate the distribution of clinical symptoms, imaging features, and long-term prognostic outcomes in patients with pulmonary nodules (PNs). The study protocol was reviewed and approved by the Institutional Review Board (IRB) of our hospital (Approval No. [2022-12-4], Approval date: 2023-01-09). Due to the observational nature of this study, the requirement for written informed consent was formally waived by the IRB, provided that all patient data were de-identified and analyzed anonymously to protect privacy.

### 2.2 Study population and enrollment period

Patients consecutive diagnosed with pulmonary nodules by chest computed tomography (CT) and admitted to the Department of Respiratory and Critical Care Medicine of our hospital between January 2023 and December 2024 were enrolled.

Pulmonary nodules were defined as single or multiple rounded/subrounded pulmonary opacities with a maximum diameter ≤30 mm, surrounded by normal lung parenchyma, without associated atelectasis, hilar enlargement, or pleural effusion, in accordance with the definition recommended by the Fleischner Society and current clinical diagnostic criteria for pulmonary nodules (15, 16).

### 2.3 Inclusion criteria

Patients were enrolled if they met all of the following criteria:

1. Age ≥18 years old;
2. Definite diagnosis of pulmonary nodule(s) on chest thin-layer CT (≤1 mm slice thickness) with complete imaging data;
3. Complete baseline clinical information, including demographic characteristics, medical history, clinical symptoms, smoking history, occupational exposure history, and comorbidities;
4. Expected and planned clinical follow-up duration of at least 24 months after initial diagnosis (patients who failed to complete the 24-month surveillance were defined as loss to follow-up and excluded from the final prognostic analysis);
5. Final nodule outcomes confirmed either by postoperative pathological examination, percutaneous puncture biopsy, or comprehensive clinical and radiological follow-up results.

### 2.4 Exclusion criteria

Patients were excluded if they met any of the following criteria:

1. Previous history of malignant tumor, especially extrapulmonary malignancy with suspected lung metastasis;
2. Nodule diameter >30 mm (classified as pulmonary mass rather than nodule);
3. Severe organic dysfunction including end-stage heart, liver, renal, or hematological diseases;
4. Incomplete clinical, imaging, pathological, or follow-up data;
5. Lost to follow-up within 24 months after initial diagnosis;
6. Concurrent active pulmonary tuberculosis, severe pulmonary infection, interstitial lung disease, or other diffuse lung diseases interfering with nodule evaluation.

### 2.5 Data collection

Baseline and follow-up data were collected from the electronic medical record system, radiology picture archiving and communication system, and outpatient follow-up records.

Collected variables included:

#### 2.5.1 General demographic characteristics

Age, gender, body mass index (BMI), smoking history (former smoker, non-smoker), etc.

#### 2.5.2 Clinical symptoms

Presence and type of clinical symptoms at initial diagnosis were recorded in detail:

- Asymptomatic (incidental detection during physical examination or other disease screening);
- chronic cough, hemoptysis, chest pain, chest tightness, checklist individual strength subscale fatigue;

Symptoms were classified as asymptomatic or symptomatic for subgroup analysis.

#### 2.5.3 Chest CT imaging features

All CT images were independently evaluated by two senior radiologists with more than 5 years of thoracic imaging experience, blinded to pathological and prognostic results. The following parameters were recorded:

- Number of nodules: solitary pulmonary nodule (SPN), multiple pulmonary nodules (MPN);
- Nodule density: solid nodule (SN), subsolid nodule (SSN), pure ground-glass nodule (pGGN), mixed ground-glass nodule (mGGN);
- Malignancy-associated imaging signs: lobulation, spiculation, pleural indentation, vascular convergence.

### 2.6 Follow-up protocol and prognostic endpoints

#### 2.6.1 Follow-up strategy

Follow-up methods included outpatient reexamination, inpatient review, telephone follow-up, and electronic medical record review.

Routine follow-up schedule:

- First reexamination: 3 months after initial diagnosis;
- Subsequent reexaminations: every 6–12 months.

#### 2.6.2 Prognostic endpoints

The prognostic endpoints were defined as follows:

- Disease progression: nodule enlargement (during the follow-up period, the maximum diameter of nodules increased by more than 2 mm), malignant transformation, local invasion, lymph node metastasis, distant metastasis, pure ground glass nodules(the average diameter increased by more than 1.5 mm, or showed realistic components), part of solid nodules(growth is defined as the average increase of nodules or solid components greater than 1.5 mm) (17, 18);
- Progression-free survival (PFS): the time from initial diagnosis to the first documented disease progression or all-cause death, whichever occurred first. Patients without either event were censored at the date of last valid follow-up.

### 2.7 Volume doubling time (VDT) calculation and image analysis

Nodule volume and volume doubling time (VDT) were quantitatively analyzed based on chest thin-section CT images (slice thickness ≤1 mm). All pulmonary nodules were segmented semi-automatically using professional post-processing software, and the contour boundaries were manually revised by two experienced radiologists to exclude peripheral blood vessels, bronchial structures, and normal lung parenchyma.

The modified Schwartz formula was applied to calculate VDT:

$$\mathrm{VDT}=(T\times \ln\;2)/\ln\;(V_{2}/V_{1})$$

In this formula, *T* represents the time interval (days) between baseline and follow-up CT examinations; *V*_1_ refers to the baseline nodule volume; and *V*_2_ represents the follow-up nodule volume.

To avoid measurement noise and ensure calculation reliability, VDT was only calculated for nodules with a relative volume increase of ≥25% during follow-up. Nodules with stable volume, decreased volume, or a volume change of less than 25% were defined as non-growing nodules and were not assigned VDT values. All image measurements were performed blindly to clinical information and pathological results to reduce assessment bias.

### 2.8 Statistical analysis

SPSS26.0 (IBM, Armonk, New York, USA) was used for all statistical analysis.

- Measurement data were tested for normality using the Kolmogorov–Smirnov test.
- The continuous variables of normal distribution are expressed by mean standard deviation (SD) and compared by independent-samples *T*-test;
- Continuous variables with non-normal distribution are expressed as median (interquartile interval, IQR) and compared by Mann–Whitney *U* test.
- Classification variables were expressed by the number of cases (percentage, %), and were compared by Chi-squared test or Fisher exact test when appropriate.

Potential prognostic factors were first screened by univariate Cox proportional hazard regression. Then the variables are further included in multivariate Cox regression analysis to determine the independent risk factors affecting the prognosis. The statistical significance was set as *P* < 0.05.

## 3 Results

### 3.1 General characteristics

**Figure 1:**
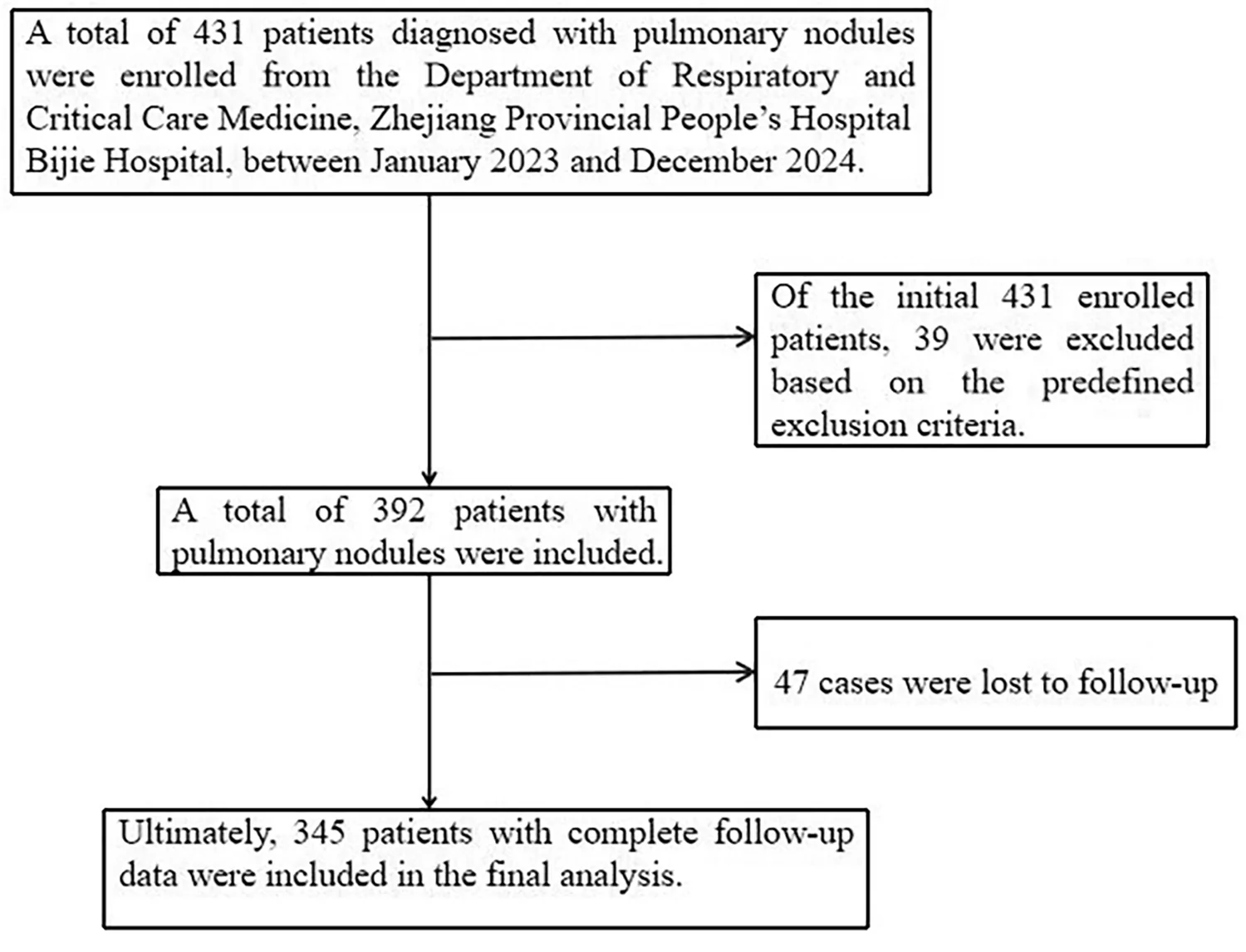
Study flowchart.

A total of 392 patients with pulmonary nodules were enrolled in the present study (Figure 1). Their demographic, clinical, radiological and follow-up outcome data are summarized below. The sex distribution of participants was balanced: 201 males (51.3%) and 191 females (48.7%). Age showed a non-normal distribution, with a median of 49.5 years and an interquartile range (IQR) of 17.0 years. Non-smokers constituted the majority of the cohort, consisting of 244 never-smokers (62.4%) and 147 former smokers (37.6%). The mean body mass index (BMI) of the study population was 23.0 ± 3.0 kg/m^2^, which fell within the normal weight range. The mean quantitative parameters were 70.5 ± 19.0 for the Checklist Individual Strength Fatigue subscale (CIS-Fatigue). Among all 392 patients, 239 (61.0%) had a prior history of COVID-19 infection, whereas 153 (39.0%) had no confirmed COVID-19 infection, indicating that over three-fifths of subjects experienced COVID-19. Only 109 patients (27.8%) presented without respiratory symptoms. Cough was the most common symptom, reported in 219 patients (55.9%). The prevalence of chest tightness, chest pain and hemoptysis was relatively low, at 5.4% (21 cases), 9.7% (38 cases) and 1.3% (5 cases), respectively (Table 1).

**Table 1:** Baseline characteristics of the patients with pulmonary nodules.

Parameter	*N*	Proportion (%)	Mean(SD)/median (IQR)
Gender	392	—	—
Male	201	51.3	—
Female	191	48.7	—
Age (year), median (IQR)	392	—	49.5 (17.0)
Smoking status	–	—	—
Non-smokers	244	62.4	—
Former smokers	147	37.6	—
BMI (Kg/m^2^), mean (SD)	392	—	23.0 (3.0)
CIS Fatigue, mean (SD)	–	—	70.5 (19.0)
Post-COVID-19 infection status	392	—	—
Yes	239	61.0	—
No	153	39.0	—
Nodule count classification	392	—	—
Solitary	193	49.2	—
Multiple	199	50.8	—
Clinical symptoms	392	—	—
Asymptomatic	109	27.8	—
Cough	219	55.9	—
Chest tightness	21	5.4	—
Chest pain	38	9.7	—
Hemoptysis	5	1.3	—
Morphology and margin	392		
Smooth and regular	260	66.3	
Lobulated and spiculated	132	33.7	
Density classification	392	—	—
Solid nodule	152	38.8	
pGGN	149	38	
mGGN	91	23.2	—
Clinical outcome	392	—	—
Stable group	246	62.8	—
Progressive group	99	25.2	—
Lost to follow-up	47	12	—

IQR, Interquartile Range; SD, Standard Deviation; BMI, Body Mass Index; CIS Fatigue, Checklist Individual Strength-Fatigue subscale; *N* = total number of patients; pGGN, Pure Ground-Glass Nodule; mGGN, Mixed Ground-Glass Nodule.

### 3.2 Imaging features of pulmonary nodules

A total of 193 patients (49.2%) had solitary pulmonary nodules, while 199 patients (50.8%) had multiple pulmonary nodules, indicating comparable proportions of single and multiple lesions. With regard to nodular margin morphology, smooth and regular nodule margins were observed in 260 patients (66.3%), whereas 132 patients (33.7%) presented nodules with high-risk malignant radiological signs, including lobulation and spiculation. According to nodule density classification, solid nodules and pure ground-glass nodules (pGGNs) accounted for similar proportions, at 38.8% (152 cases) and 38.0% (149 cases), respectively. Part-solid (mixed ground-glass) nodules made up the lowest proportion at 23.2% (91 cases) (Table 1).

### 3.3 Distribution of follow-up outcomes

A total of 392 patients were enrolled in this study. During follow-up, nodules remained stable in the majority of participants (246 cases, 62.8%), while nodule progression was observed in 99 patients (25.2%). Forty-seven patients (12.0%) were lost to follow-up due to failure to establish contact (Table 1). Table 2 summarized the comparison of clinical symptoms and quantitative parameters between the stable nodule group and progression group. The median age was 48.8 years (IQR = 8.9) in the stable group and 50.6 years (IQR = 7.8) in the progression group, with no statistically significant difference observed between subgroups (*P* = 0.053). The mean BMI was 22.5 ± 2.3 kg/m^2^ for patients with stable nodules and 23.2 ± 3.5 kg/m^2^ for patients with progressive nodules, and the difference failed to reach statistical significance (*P* = 0.102). Statistically significant differences were detected in volume doubling time (VDT) and CIS-Fatigue scores. The mean VDT value was markedly higher in the stable group (569.8 ± 103.5) than that in the progression group (386.5 ± 98.6, *P* = 0.023). For fatigue status, the progression group exhibited substantially elevated CIS-Fatigue scores (85.6 ± 17.8) relative to the stable group (56.8 ± 18.5, *P* = 0.015). Although the progression group exhibited significantly higher baseline CIS-Fatigue scores compared with the non-progression group (*P* < 0.05), CIS-Fatigue was not a predictive factor for pulmonary nodule progression. Regarding former smoking status, 52 former smokers were identified in the stable group and 92 former smokers in the progression group, and the distribution of previous smoking history showed no significant intergroup difference (*P* = 0.235). Collectively, VDT and CIS-Fatigue scores were the only two quantitative indicators that differed significantly between the two subgroups. Age, BMI and history of former smoking showed no statistically meaningful disparities when comparing patients with stable vs. progressive pulmonary nodules.

**Table 2:** Characteristics of stable group and progression group in cohort.

Parameter	Stable group	Progression group	*P*-value
Age (year), median (IQR)	48.8 (8.9)	50.6 (7.8)	0.053
BMI (Kg/m^2^), mean(SD)	22.5 (2.3)	23.2 (3.5)	0.102
VDT, mean (SD)	569.8 (103.5)	386.5 (98.6)	0.023*
CIS Fatigue, mean (SD)	56.8 (18.5)	85.6 (17.8)	0.015*
Former smokers, median (IQR)	52 (5.2)	92 (6.5)	0.235

IQR, interquartile range; SD, standard deviation; BMI, body mass index; VDT, volume doubling time; CIS fatigue, checklist individual strength subscale fatigue.

* *P* < 0.05, statistically significant.

### 3.4 Prognostic determinants of pulmonary nodules

For this prospective cohort study, univariate Cox proportional hazard regression analysis was performed to screen potential risk factors associated with the progression of pulmonary nodules, including age (HR = 1.103), smoking status (HR = 1.157), post-COVID-19 infection status (HR = 1.214), and nodule morphology and margin (HR = 2.497) (Table 3). These variables were further incorporated into multivariate Cox regression analysis to identify independent risk factors affecting nodule progression. The results demonstrated that age, smoking status, and nodule morphology and margin were independent risk factors for nodule progression in patients with pulmonary nodules. Each 5-year increment in age was associated with a 1.2-fold higher risk of nodule growth. Former smokers exhibited a 1.2-fold greater risk of pulmonary nodule progression compared with never-smokers (HR = 1.210). Nodule morphology and margin were strongly correlated with nodule progression. The risk of growth for nodules with lobulated and spiculated margins was approximately 1.5 times that of nodules with smooth and regular margins (Table 4).

**Table 3:** Univariate Cox regression analysis of factors associated with pulmonary nodule progression.

Variables	HR	*P*-value	95% CI for HR	
			Lower limit	Upper limit
Age (every year)	1.103	0.019*	1.004	1.243
Gender	0.990	0.058	0.981	1.098
BMI	0.987	0.321	0.753	1.023
Smoking Status	1.157	0.048*	0.987	1.515
Post-COVID-19 infection status	1.214	0.003*	1.074	1.394
CIS Fatigue	0.997	0.712	0.952	1.665
Nodule Count Classification	1.105	0.081	1.004	1.425
Morphology and Margin	2.497	0.001*	1.490	4.186
Density Classification	1.015	0.058	0.963	1.352

HR, hazard ratio; CI, confidence interval.

* *P* < 0.05, statistically significant.

**Table 4:** Multivariate cox regression analysis of independent risk factors for pulmonary nodule progression.

Variables	HR	*P*-value	95% CI for HR	
			Lower limit	Upper limit
Age (every 5 years)	1.208	0.033*	0.956	1.643
Smoking status	1.210	0.044*	0.894	2.035
Morphology and margin	1.514	0.025*	1.105	3.759

HR, hazard ratio; CI, confidence interval.

* *P* < 0.05, statistically significant.

## 4 Discussion

This prospective cohort study enrolled 392 patients with pulmonary nodules to systematically analyze their demographic, clinical profiles, radiological characteristics and long-term follow-up outcomes. Univariable and multivariable Cox proportional hazard regression models were constructed to screen independent prognostic predictors of nodule progression. The real-world cohort data can provide evidence for clinical risk stratification, individualized surveillance strategy formulation and mechanism exploration of pulmonary nodules, and deliver practical guidance for standardized management of pulmonary nodules populations.

The cohort was predominantly composed of middle-aged and young patients with a median age of 49.5 years, and more than 60% of participants were under 55 years old. This age distribution is consistent with the latest epidemiological investigations of pulmonary nodules in domestic and foreign populations, which have demonstrated a rising detection rate of pulmonary nodules among young and middle-aged individuals in recent years (19). The trend is largely attributable to the widespread popularization of low-dose computed tomography (LDCT) screening, improved public health awareness, as well as residual pulmonary inflammatory changes secondary to respiratory viral infections, including COVID-19 (20–27). In terms of gender composition, no significant difference was observed in the overall nodule incidence between male and female patients in our cohort, which differs slightly from traditional studies that reported a higher prevalence in males with long-term smoking exposure (28). The balanced gender distribution in this study may be explained by the increased proportion of non-smoking-related inflammatory nodules and benign proliferative lesions in post-epidemic pulmonary nodule populations. Notably, most participants were non-smokers with normal BMI, overturning the traditional cognition that pulmonary nodules mainly affect elderly heavy smokers (7). This indicates nodule formation is modulated by viral infection, immune status and environmental factors, rather than aging and smoking alone (29).

Symptom analysis showed that cough was the most common symptom (55.9%), while nearly one-third of patients were completely asymptomatic, with rare severe respiratory symptoms including chest pain and hemoptysis. This aligns with the occult clinical characteristics of low-risk pulmonary nodules, whereby indolent inflammatory or adenomatous lesions lack specific manifestations and are easily missed without regular CT screening (30). Thus, routine low-dose CT surveillance is essential for asymptomatic COVID-19 convalescents to avoid delayed diagnosis of progressive nodules.

We also evaluated nodule volumetric dynamics using volume doubling time (VDT). Only nodules with ≥25% relative volume growth during follow-up were assigned VDT values; stable, shrinking, or minimally changing lesions were classified as non-growing. In our cohort, shorter VDT was associated with lobulated or spiculated margins and predicted higher progression risk, reinforcing that both static morphology and dynamic volumetric change improve risk discrimination beyond baseline imaging alone.

The present prospective cohort study explored risk factors for pulmonary nodule progression using Cox regression models. Multivariate analysis confirmed that older age, smoking history, and irregular nodule morphology with lobulated or spiculated margins were independent risk factors for nodule progression (31). Age was an independent non-modifiable predictor, with every 5-year increase in age elevating the risk of nodule progression by 1.2-fold. Advanced age is associated with impaired pulmonary immune surveillance and cumulative chronic inflammatory injury, which facilitates abnormal cell proliferation and persistent nodule growth (32). Accordingly, elderly patients should be regarded as a high-risk group requiring intensified imaging follow-up, which aligns with the current lung nodule screening guidelines (33). Smoking status was also an independent predictor of nodule progression in this cohort. Former smokers exhibited a 1.2-fold higher risk of nodule progression compared with never-smokers. Notably, smoking status showed no significant intergroup difference in unadjusted baseline comparison, indicating that its prognostic effect was masked by other baseline confounding factors and could only be identified after multivariate adjustment in the Cox model. Long-term tobacco exposure triggers persistent oxidative stress and irreversible injury to airway epithelium, facilitating continuous proliferation of pulmonary lesions even after smoking cessation (17, 34, 35). This finding further suggests that smoking history should be integrated into routine risk stratification of pulmonary nodules to refine long-term surveillance strategies. Among all predictors evaluated in the multivariate model, nodule morphology and margin characteristics demonstrated the strongest prognostic performance. Nodule morphological and marginal features demonstrated the strongest predictive efficacy in the multivariate model. Nodules with lobulated and spiculated margins had approximately 1.5-fold higher progression risk than nodules with smooth and regular boundaries. These typical imaging signs reflect aggressive biological behaviors, including uneven lesion growth and peripheral interstitial infiltration, which are well-validated high-risk indicators for nodule progression and malignant transformation (19, 36). Notably, post-COVID-19 infection was significant only in univariate analysis and failed to maintain statistical significance after multivariate adjustment. It is speculated that COVID-19-associated pulmonary inflammation may induce transient imaging abnormalities but cannot independently drive long-term nodule progression (37–39). In conclusion, age, smoking history, and irregular nodule morphology are reliable independent risk factors for pulmonary nodule progression. These findings provide evidence for precise risk stratification and individualized follow-up management of patients with pulmonary nodules.

This study has limitations. First, 47 of the initial 392 patients were lost to follow-up and excluded from all prognostic analyses; we did not perform a separate baseline comparison for the lost-to-follow-up subgroup because these patients were not incorporated into the final analysis. Attrition may introduce selection bias, and our conclusions apply only to patients who completed 24-month surveillance. Second, this was a single-center prospective cohort, and residual confounding cannot be fully excluded despite multivariable adjustment. Third, VDT was only calculated for growing nodules, so we cannot compare VDT across the entire cohort. Larger multicenter studies with centralized imaging review and standardized follow-up are needed to validate these predictors. Fourth, this study did not adopted a composite progression endpoint that encompasses heterogeneous events, including nodule enlargement, newly emerged solid components, malignant transformation, local invasion, and distant metastasis. These outcomes vary considerably in biological severity and clinical implications, ranging from early indolent imaging changes to aggressive malignant progression. This heterogeneity may affect result interpretation: the identified prognostic factors predict overall nodule progression events rather than specifically invasive or metastatic malignant progression. Therefore, the predictive values of these clinical and imaging indicators should not be overinterpreted as exclusive biomarkers for advanced lung cancer. Larger multicenter studies with unified and hierarchical endpoint definitions are needed to further validate these predictors.

In conclusion, age, smoking history, irregular nodule morphology, and volumetric growth reflected by shorter VDT are reliable risk markers for pulmonary nodule progression. Combined assessment of baseline clinical features, static imaging signs, and longitudinal volumetric change can support precise risk stratification and individualized follow-up management.

## 5 Conclusion

In this prospective cohort study involving 392 patients with pulmonary nodules, we found that shortened volume doubling time and increased fatigue scores were significantly associated with nodule progression. After multivariate adjustment, age, smoking history, and nodule margins with lobulation and spiculation were confirmed as independent risk factors for nodule growth. Every 5-year increase in age and previous smoking elevated the risk of nodule progression by 1.2 times. Nodules with malignant morphological features had approximately 1.5 times higher progressive risk than those with smooth edges. Although post-COVID-19 infection was correlated with nodule progression in univariate analysis, it was not an independent prognostic factor. Clinicians can comprehensively evaluate patients according to age, smoking history and CT imaging characteristics to carry out precise risk stratification. For patients with the above high-risk factors, intensified regular CT surveillance is recommended to dynamically monitor changes in pulmonary nodules.

## Data Availability

The raw data supporting the conclusions of this article will be made available by the authors, without undue reservation.
